# Infarct Growth Rate Predicts Early Neurological Improvement in Ischemic Stroke After Endovascular Thrombectomy

**DOI:** 10.3390/brainsci15030303

**Published:** 2025-03-13

**Authors:** Zhihang Huang, Shuaiyu Chen, Bin Wei, Yan E, Jingwen Qi, Xiaohao Zhang, Teng Jiang

**Affiliations:** Department of Neurology, Nanjing First Hospital, Nanjing Medical University, Nanjing 210006, China; huangzhihang@stu.njmu.edu.cn (Z.H.); chensy1997@stu.njmu.edu.cn (S.C.); weibin2020nj@163.com (B.W.); eyan@njmu.edu.cn (Y.E.); qijingwen@stu.njmu.edu.cn (J.Q.); zhangxiaohao@njmu.edu.cn (X.Z.)

**Keywords:** IGR, large vessel occlusion, mechanical thrombectomy, neurological improvement

## Abstract

Background and Purpose: The infarct growth rate (IGR) is a major modifier of the therapeutic effect of endovascular thrombectomy. The objective of this paper was to explore the utility of the IGR measured by perfusion the imaging in predicting early neurological improvement (ENI) of patients treated with EVT. Methods: We reviewed consecutive large vessel occlusive stroke in the anterior circulation and treated by thrombectomy between October 2019 to July 2024. The IGR was defined as the ischemic core volume (apparent diffusion coefficient ≤ 620 × 10^−6^ mm^2^/s or relative cerebral blood flow < 30%) divided by the time from stroke onset to imaging. ENI was defined as a reduction ≥ 6 points in the NIH Stroke Scale score at 24 h after the procedure, or an NIH Stroke Scale score of 0 or 1 on day 7 of hospitalization or at discharge if it occurred before day 7. Results: A total of 407 patients (mean age, 69.3 ± 12.5 years; 63.1% of male) were included, of whom 149 (36.6%) achieved ENI. Among all enrolled patients, 281 patients were classified as slow (IGR < 10 mL/h) and 126 fast progressors (IGR ≥ 10 mL/h). In multivariable analyses, fast progressors had a lower likelihood of achieving ENI after endovascular thrombectomy (odds ratio, 0.442; 95% confidence intervals, 0.269–0.729, *p* = 0.001) as compared to slow progressors. Subgroup analyses further confirmed these results. Furthermore, the odds of ENI decreased by 7.3% for each 5 mL/h increase in the IGR (odds ratio, 0.927; 95% confidence intervals, 0.875–0.982, *p* = 0.011). Conclusions: The present study found that the pre-treatment IGR was associated with ENI in thrombectomy patients.

## 1. Introduction

Endovascular thrombectomy (EVT) has been demonstrated to provide clinical benefits for patients with proximal large vessel occlusions within a 24-h therapeutic window following stroke onset [1,2]. Recanalization and subsequent reperfusion play a pivotal role in rescuing ischemic penumbra in patients with acute large vessel occlusion. Nevertheless, almost 50% of participants treated by EVT were dead or dependent at 3 months, despite successful recanalization [3]. It is well-investigated that the neurological assessment at 24 h is of high value to estimate the therapeutic response of EVT [4]. A systematic review included nine studies with 2355 patients has found that early neurological improvement (ENI) is a promising predictor of good functional outcome at 90 days and is associated with lower rates of mortality and symptomatic intracranial hemorrhage [5]. Also, data from the ETIS (Endovascular Treatment of Ischemic Stroke) registry confirmed that ENI on day 1 following EVT for basilar artery occlusion is a strong independent predictor of a favorable 3-month clinical outcome [6]. Therefore, the identification of predictors of ENI is important to determine which patients are likely to benefit from EVT and in whom futile recanalization can be avoided.

Infarct progression is highly variable even during initial hours after stroke onset, with previous research indicating that approximately 20% of patients finalize their infarcts early [7]. The infarct growth rate (IGR), as a composite metric integrating both baseline perfusion-defined infarct volume and time from stroke onset to imaging, reflects the dynamic pathophysiological progression of cerebral ischemia. This integrated parameter demonstrates superior predictive accuracy for clinical outcomes compared with either time from onset to imaging or initial infarct volume alone [8]. We did, like others, find that the IGR was associated with occlusion in internal carotid artery and poor collaterals [9,10]. A diminished IGR correlated with the existence of a target mismatch indicative of a substantial salvageable penumbra [11]. In a prospective multicenter cohort study examining imaging selection for endovascular thrombectomy, the IGR is independently correlated with functional independence [12]. Also, fast progressors had higher mortality and final infarct volume in comparison to slow progressors [12]. Moreover, data from international stroke perfusion imaging registry indicated that an IGR > 25 mL/h correlates with clinical and imaging outcomes with EVT in comparison to intravenous thrombolysis alone [8]. Nonetheless, the influence of the IGR on initial treatment response, particularly the ENI following EVT, is still unclear.

We therefore performed this study to determine whether or not the IGR can predict the ENI in patients with large vessel occlusive stroke in the anterior circulation after EVT treatment.

## 2. Materials and Methods

### 2.1. Study Design and Participants

The study cohort were selected from consecutive patients with ischemic stroke due to larger vessel occlusion in the anterior circulation (internal carotid artery, M1 or M2 segments of middle cerebral artery) who were treated by EVT in Nanjing First Hospital from October 2019 to July 2024. The inclusion criteria were as follows: (1) older than 18 years; (2) available data of pretreatment perfusion imaging. Patients treated with devices other than a stent-like retriever and aspiration system were excluded from this study. The Ethics Committee of Nanjing First Hospital approved this study (KY20240822-KS-05). The need for informed consent was waived due to this study’s retrospective nature. All study participants were fully anonymized.

### 2.2. Baseline Data Collection

We recorded the baseline clinical data including demographic characteristics, vascular risk factors, blood pressure, baseline NIH Stroke Scale (NIHSS) score and Alberta stroke program early CT score (ASPECTS), and the Trial of ORG 10172 in Acute Stroke Treatment (TOAST) classification [13]. The procedural parameters, including occlusion site, time from stroke onset to reperfusion, bridging treatment, recanalization status, and collateral circulation, were evaluated by two experienced operators and were collected for analysis. Pre-treatment collateral status was assessed using the American Society of Interventional and Therapeutic Neuroradiology/Society of Interventional Radiology grading system, with grade 0–1 representing poor collaterals, and grade 2–4 representing moderate to excellent collaterals [14]. Recanalization status was evaluated based on the modified thrombolysis in cerebral infarction (mTICI) grading system [15]. Successful recanalization was defined as an mTICI score of 2b or 3 [16,17]. The functional outcome was assessed using the modified Rankin scale.

### 2.3. Measurement of IGR

All patients performed the magnetic resonance perfusion or computed tomography perfusion before surgery. The IGR was calculated as follows: initial infarct volume/time from stroke onset to imaging acquisition [9,12,18]. Infarct volume was determined by apparent diffusion coefficient ≤ 620 × 10^−6^ mm^2^/s or relative cerebral blood flow < 30% on perfusion imaging using the automated software. For “wake-up” stroke patients, the last-known well time was used as stroke onset time. According to prior study [12], patients were classified as slow (IGR < 10 mL/h) and fast progressors (IGR ≥ 10 mL/h).

### 2.4. Definition of ENI

In accordance with previous studies [16,19], ENI was defined as a reduction ≥ 6 points in NIHSS score at 24 h after EVT, or a NIHSS score of 0 or 1 on day 7 of hospitalization or at discharge if it occurred before day 7.

### 2.5. Statistical Analysis

Data were summarized as number (%), mean ± standard deviation, or median (interquartile range [IQR]). Differences in baseline characteristics were compared using the Student t test or Mann Whitney U test and categorical variables using the χ^2^ test or Fisher exact test where appropriate. Two separate multivariable logistic regression models were performed to evaluate the ENI risk by calculating odds ratio (OR) and 95% confidence intervals (CI). Model 1 adjusted for age and sex; Model 2 additionally adjusted for variables with a *p* value < 0.1 in the univariate analysis (including successful reperfusion, stroke etiology, occlusion site, and baseline glucose levels). Furthermore, several sensitivity analyses were conducted to test the robustness of our findings. All *p* values were two-sided, and a significance level of 0.05 was considered statistically significant. All statistical analyses were performed using R software (version 4.3.1; Vienna, Austria).

## 3. Results

### 3.1. Participant Baseline Characteristics

A total of 407 consecutive subjects (mean age, 69.3 ± 12.5 years; 257 male) were included in this study. Table 1 demonstrated the detailed information about demographic characteristics, imaging and treatment characteristics of study population. The median NIHSS was 12.0 (IQR, 9.0–16.0), median core volume was 17.0 mL (IQR, 4.5–49.0), and time from onset to perfusion imaging acquisition was 4.2 h (IQR, 2.0–8.9). Intravenous thrombolysis was administered in 125 (30.7%) patients and 376 (92.4%) patients achieved complete or near-complete mechanical recanalization. Among them, 120 (29.6%) patients were “wake-up” stroke.

### 3.2. Baseline Characteristics in Fast and Slow Progressors

The median IGR was 3.6 mL/h (IQR, 0.9–13.1) with a range of 0 to 288.0 mL/h. Two hundred eighty-one patients were classified as slow (IGR < 10 mL/h) and 126 fast progressors (IGR ≥ 10 mL/h). Compared with patients in the slow progressors group, fast progressors had a lower baseline ASPECTS (median 9.0 versus 9.0, *p* = 0.001) and prevalence of “wake-up” stroke (20.6% versus 33.7%, *p* = 0.008), a higher 90-day modified Rankin scale score (median, 3.0 versus 2.0, *p* = 0.007), more severe stroke symptoms (median NIHSS score, 14.0 versus 12.0, *p* = 0.001), and prevalence of intravenous thrombolysis treatment (46.8% versus 30.6%, *p* = 0.002), poor circulation status (61.9% versus 45.2%, *p* = 0.002), and occlusion site in internal carotid artery (43.7% versus 31.1%, *p* = 0.014). The two groups had not significance difference in age, sex, vascular risk factors and cause of stroke.

### 3.3. The Effect of IGR on ENI

During hospitalization, 149 (36.6%) patients experienced ENI after EVT. Table 2 summarized the results of the comparison of baseline characteristics in patients with and without ENI. Compared with the non-ENI group, the ENI group had a higher successful reperfusion rate (97.3% versus 89.5%; *p* = 0.004) and baseline ASPECTS (mean, 9.1 versus 8.7; *p* = 0.001). Patients with ENI had lower baseline blood glucose levels (mean, 6.4 versus 7.4 mmol/L; *p* = 0.001), ischemic core volume (median, 7.7 versus 24.8 mL; *p* = 0.001), IGR (median, 1.7 versus 6.1 mL/h; *p* = 0.001), and 90-day modified Rankin scale score (median, 0 versus 3.0, *p* = 0.001). Moreover, the ENI group exhibited a decreased prevalence of large artery atherosclerosis (40.9% versus 56.2%, *p* = 0.008) and occlusion site in ICA (28.2% versus 38.9%, *p* = 0.029).

After multivariable adjustment for potential confounders, including demographic characteristics, successful reperfusion, stroke etiology, occlusion site, and baseline glucose levels), fast progressors had a lower likelihood of achieving ENI after EVT (OR, 0.442; 95% CI, 0.269–0.729, *p* = 0.001) as compared to slow progressors. The observed association remained prominent when the IGR was analyzed as a continuous variable. Furthermore, the odds of ENI decreased by 7.3% for each 5 mL/h increase in the IGR (OR, 0.927; 95% CI, 0.875–0.982, *p* = 0.011; Table 3).

**Figure 1:**
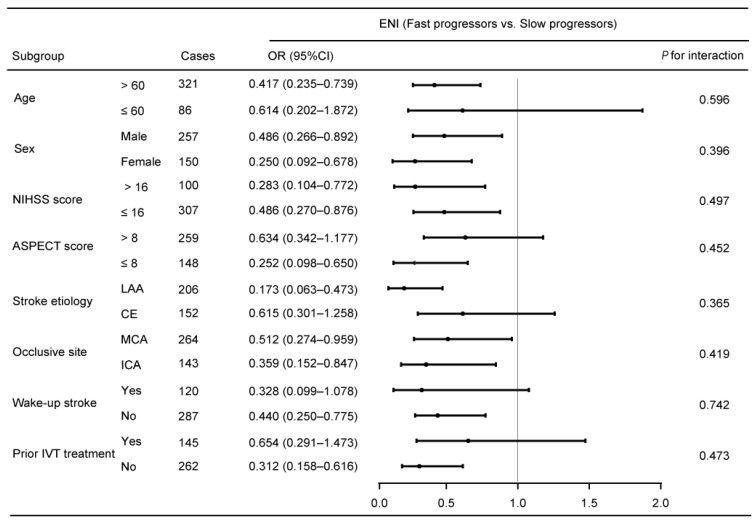
Subgroup analyses of slow and fast progressors in relation to ENI. Odds ratio is calculated after adjustment for the same variables as model 2 in Table 3, except for the stratified variable. ASPECTS, the Alberta Stroke Program Early Computed Tomography Score; CI, confidence interval; ENI, early neurological improvement; IVT, intravenous thrombolysis; OR, odds ratio; NIHSS, National Institute of Health Stroke Scale.

In addition, the association of the IGR (fast versus slow progressors) with the odds of ENI was similar across subgroups stratified according to age, sex, baseline NIHSS score, pre-treatment ASPECTS, stroke etiology, prior intravenous thrombolysis treatment, occlusion site and “wake-up” stroke (all *p* > 0.05 for interaction; Figure 1).

## 4. Discussion

In this study of patients with large vessel occlusive stroke in anterior circulation who were treated by EVT, we investigated whether the pre-treatment IGR was correlated with ENI. We determined that an elevated IGR was independently correlated with the reduced likelihood of attaining ENI, even after adjusting for potential confounders. Our findings may have potential implications for the triage of treatment, prognostication, and the identification of patient populations in large artery occlusive stroke cases for future neuroprotective trials.

We found that 149 (36.6%) patients obtained ENI during hospitalization, which is notably lower than that reported in a multi-center stroke data bases at the University Medical Center Hamburg-Eppendorf and Stanford University (26.0%) [20], and is similar to a multicenter registry study in China (38.9%) [16]. Furthermore, ENI incidence in our study was significantly lower than that reported in the randomized controlled trials of DAWN study (48.0%) [19], whose were data obtained from populations of white dominance. This may be ascribed to the higher prevalence of cerebral atherosclerosis in the Asian population. An atherosclerotic occlusion may be more challenging to recanalize than a cardioembolic occlusion, as the former was commonly accompanied with a significant stenosis. Multiple passes with a thrombectomy device for atherosclerotic occlusion may cause vascular endothelial damage and extend the duration of the procedure, which are associated with adverse clinical outcomes [21]. Also, the broadened indications, limited experiences, and unfavorable treatment conditions may result in a diminished likelihood of ENI in real-world practices.

Previous studies have associated ENI with a younger age, reduced initial NIHSS scores, decreased ischemic core volumes at presentation, successful reperfusion, poor collateral circulation, higher time from stroke onset to recanalization, and lower blood glucose levels [6,16,20,22,23]. Ichijo et al. found that the development of leptomeningeal collaterals seems to play a pivotal role in obtaining ENI and that the reversion of arterial collaterals predicts a favorable clinical outcome after treatment [22]. In addition, a study conducted by Pu et al. revealed that ENI (characterized by a 42% reduction in the NIHSS score from the baseline within 7 days post-EVT) was linked to poor arterial collateral status [23]. The results of these investigations contradict our findings, as we did not observe a significant link between collateral circulation status and ENI following EVT. Nonetheless, we note that differing definitions of ENI may restrict direct comparisons of our results with those of other investigations.

Since initial stroke assessments and thrombectomy triage are predominantly performed by either the ASPECTS or perfusion imaging, we evaluated the impact of the IGR on early therapeutic response in patients receiving EVT. Sarraj et al. divided subjects according to the IGR into slow (IGR under 10 mL/h) and fast (IGR above 10 mL/h) progressors in the secondary analysis of SELECT study, and found that the IGR independently correlated with functional independence, mortality, and final infarct volume after EVT [12]. However, this study did not detect the influence of IGR on the therapeutic response after EVT. Our study extended previous findings and further indicated better neurological improvement in individuals with slow progression as compared to fast progression. In this study, elevated baseline blood glucose levels were found in fast progressors, which was similar to the prior research [12]. Hyperglycemia compromises the blood–brain barrier by various biological processes, including the induction of inflammatory responses, oxidative stress, excitatory chemokines, and directly attacking the neurovascular unit [24], all of which may prevent the ENI after recanalization. Moreover, 43.7% of individuals exhibiting rapid progression had occlusion in the internal carotid artery, compared to 31.1% in the slow progressor group. Patients with internal carotid artery occlusion have lower reperfusion rates, lower intravenous thrombolysis rates, and a more complicated procedure when attempting to open the vessel [25,26]. Additionally, consistent with prior studies [12,27], our investigation revealed that collateral status was another factor of the IGR. Relatively poorer collateral circulation may diminish perfusion to brain tissue in oligemic areas, thereby exacerbating cerebral ischemic damage [28].

The present study has several potential limitations. Firstly, the sample size of some of our subgroups was relatively small, resulting in low statistical power to uncover significant differences. Secondly, ischemic core volume was measured by the software of RAPID (rapid processing of perfusion and diffusion; iSchemaView, Menlo Park, CA, USA) and F-STROKE (Fast-processing of ischemic stroke software; Version 1.0; Neuroblem Ltd. Company, Shanghai, China) in this study, which might introduce software-related variability. However, F-STROKE was confirmed to have an excellent agreement with the widely used analysis tool of RAPID in measuring the ischemic core volume and penumbra volume [29]. Another limitation is that some advances in devices and techniques of thrombectomy used during our 5-year study period may have affected our results. Fourthly, the inclusion of “wake-up” stroke patients creates inherent selection bias as this subgroup may not fully represent the broader stroke population. Finally, the observed IVT rate in our cohort was relatively lower compared to other study populations, which may weaken the generalizability of our findings. Prospective studies with a large sample are warranted to confirm our results.

## 5. Conclusions

In summary, this study demonstrated that the pre-treatment IGR is a significant predictor of ENI in patients receiving EVT. Our results suggested that the IGR may help to identify the patients who have the potential to respond favorably to EVT treatment. These findings carry potential implications for both the design of neuroprotection trials and the refinement of triage protocols in primary stroke centers.

## Figures and Tables

**Table 1:** Comparison of baseline characteristics between slow and fast progressors.

Variables	All Patients (n = 407)	Fast Progressors (n = 126)	Slow Progressors (n = 281)	*p* Value
Demographic characteristics				
Age, years	69.3 ± 12.5	68.6 ± 13.4	69.6 ± 12.2	0.462
Male, n (%)	257 (63.1)	85 (67.5)	172 (61.2)	0.227
Vascular risk factors, n (%)				
Hypertension	300 (73.7)	94 (74.6)	206 (73.3)	0.784
Diabetes mellitus	139 (34.2)	50 (39.7)	89 (31.7)	0.115
Hyperlipidemia	52 (12.8)	15 (11.9)	37 (13.2)	0.724
Smoking	164 (40.3)	52 (41.3)	112 (39.9)	0.788
Coronary heart disease	64 (15.7)	21 (16.7)	43 (15.3)	0.727
Systolic blood pressure, mmHg	141.1 ± 22.2	140.6 ± 22.6	141.3 ± 20.1	0.766
Diastolic blood pressure, mmHg	84.8 ± 13.6	85.0 ± 14.3	84.8 ± 13.3	0.850
Time from onset to perfusion imaging acquisition, h	4.2 (2.0, 8.9)	2.2 (1.3, 4.0)	5.7 (2.8, 10.8)	0.001
Ischemic core volume, mL	17.0 (4.5, 49.0)	61.0 (33.9, 110.5)	8.7 (2.9, 20.5)	0.001
IGR, mL/h	3.6 (0.9, 13.1)	23.9 (14.3, 46.4)	1.7 (0.5, 4.0)	0.001
Early neurological improvement, n (%)	149 (36.6)	31 (24.6)	118 (42.0)	0.001
Baseline NIHSS, score	12.0 (9.0, 16.0)	14.0 (10.0, 18.0)	12.0 (8.0, 16.0)	0.001
Baseline ASPECTS, score	8.8 ± 1.1	8.5 ± 1.2	9.0 ± 1.0	0.001
Prior anticoagulant therapy, n (%)	59 (14.5)	20 (15.9)	39 (13.9)	0.597
“Wake-up” stroke, n (%)	120 (29.6)	26 (20.6)	94 (33.7)	0.008
Stroke subtypes, n (%)				0.067
Large artery atherosclerosis	206 (50.6)	53 (42.1)	153 (54.4)	
Cardiac embolism	152 (37.3)	56 (44.4)	96 (34.2)	
Others/unknown	49 (12.1)	17 (13.5)	32 (11.4)	
Prior IVT treatment, n (%)	145 (35.6)	59 (46.8)	86 (30.6)	0.002
Poor collateral circulation, n (%)	205 (50.4)	78 (61.9)	127 (45.2)	0.002
Successful reperfusion, n (%)	376 (92.4)	117 (92.9)	259 (92.2)	0.809
Emergency stent implantation, n (%)	53 (13.0)	21 (16.7)	32 (11.4)	0.143
Time from onset to revascularization, h	6.3 (4.0, 11.1)	4.3 (3.2, 6.3)	7.9 (4.7, 12.2)	0.001
Occlusive site, n (%)				0.014
Internal carotid artery	142 (35.0)	55 (43.7)	87 (31.1)	
Middle cerebral artery	264 (65.0)	71 (56.3)	193 (68.9)	
mRS score at 90 days, score	2.0 (0, 4.0)	3.0 (1.0, 5.0)	2.0 (0, 4.0)	0.007
Laboratory data				
Baseline blood glucose levels, mmol/L	7.0 ± 2.4	7.3 ± 2.3	6.9 ± 2.4	0.115
Hs-CRP levels, mg/L	10.9 (4.0, 26.4)	12.6 (3.6, 21.4)	8.9 (4.4, 26.9)	0.783

Abbreviations: ASPECTS, the Alberta Stroke Program Early Computed Tomography Score; IGR, Infarct growth rate; Hs-CRP, Hyper-sensitive C-reactive protein; IVT, Intravenous thrombolysis; mRS, modified Rankin Scale; NIHSS, National institute of health stroke scale.

**Table 2:** Comparison of baseline characteristics in patients with and without ENI after EVT.

Variables	With ENI (n = 149)	Without ENI (n = 258)	*p* Value
Demographic characteristics			
Age, years	68.2 ± 12.8	70.0 ± 12.3	0.151
Male, n (%)	101 (67.8)	156 (60.5)	0.140
Medical history, n (%)			
Hypertension	103 (69.1)	197 (76.4)	0.110
Diabetes mellitus	44 (29.5)	95 (36.8)	0.135
Hyperlipidemia	20 (13.4)	32 (12.4)	0.767
Smoking	65 (43.6)	99 (38.4)	0.298
Coronary heart disease	28 (18.8)	36 (14.0)	0.196
Systolic blood pressure, mmHg	139.1 ± 21.5	142.3 ± 22.6	0.168
Diastolic blood pressure, mmHg	84.5 ± 14.3	85.1 ± 13.2	0.666
Time from onset to perfusion imaging acquisition, h	4.0 (2.1, 8.9)	4.6 (1.9, 8.9)	0.873
Ischemic core volume, mL	7.7 (2.9, 23.0)	24.8 (8.9, 61.0)	0.001
IGR, mL/h	1.7 (0.5, 6.9)	6.1 (1.6, 16.7)	0.001
Baseline NIHSS, score	13.0 (9.0, 17.0)	12.0 (8.0, 16.0)	0.158
Baseline ASPECTS, score	9.1 ± 0.9	8.7 ± 1.2	0.001
Prior anticoagulant therapy, n (%)	20 (13.4)	39 (15.1)	0.640
“Wake-up” stroke, n (%)	39 (26.2)	81 (31.6)	0.245
Stroke subtypes, n (%)			0.008
Large artery atherosclerosis	61 (40.9)	145 (56.2)	
Cardiac embolism	64 (43.0)	88 (34.1)	
Others/unknown	24 (16.1)	25 (9.7)	
Prior IVT treatment, n (%)	55 (36.9)	90 (34.9)	0.681
Poor collateral circulation, n (%)	71 (47.7)	134 (51.9)	0.405
Successful reperfusion, n (%)	145 (97.3)	231 (89.5)	0.004
Emergency stent implantation, n (%)	16 (10.7)	32 (14.3)	0.298
Time from onset to revascularization, h	6.2 (3.7, 11.5)	6.3 (4.2, 10.9)	0.606
Occlusive site, n (%)			0.029
Internal carotid artery	42 (28.2)	100 (38.9)	
Middle cerebral artery	107 (71.8)	157 (61.1)	
mRS score at 90 days, score	0 (0, 2.0)	3.0 (1.0, 5.0)	0.001
Laboratory data			
Baseline blood glucose levels, mmol/L	6.4 ± 1.9	7.4 ± 2.5	0.001
Hs-CRP levels, mg/L	8.2 (3.5, 23.0)	12.7 (4.2, 28.4)	0.249

Abbreviations: ASPECTS, the Alberta Stroke Program Early Computed Tomography Score; IGR, infarct growth rate; Hs-CRP, hyper-sensitive C-reactive protein; IVT, intravenous thrombolysis; mRS, modified Rankin Scale; NIHSS, National Institute of Health Stroke Scale.

**Table 3:** Multivariate regression analysis for the correlation between the IGR and ENI.

Variables	Unadjusted Model		Model 1		Model 2	
	OR (95% CI)	*p* Value	OR (95% CI)	*p* Value	OR (95% CI)	*p* Value
IGR (as continuous variable)	0.986 (0.975–0.997)	0.011	0.985 (0.974–0.996)	0.008	0.985 (0.973–0.996)	0.010
IGR, per 5 mL/h increase	0.936 (0.886–0.989)	0.018	0.934 (0.884–0.986)	0.014	0.927 (0.875–0.982)	0.011
IGR						
Slow progressors	Reference		Reference		Reference	
Fast progressors	0.451 (0.282–0.721)	0.001	0.434 (0.270–0.698)	0.001	0.442 (0.269–0.729)	0.001

Abbreviations: CI, confidence interval; IGR, infarct growth rate; OR, odds ratio. Model 1: adjusted for demographic characteristics. Model 2: adjusted for demographic characteristics, successful reperfusion, stroke etiology, occlusion site, and baseline glucose levels.

## Data Availability

The data presented in this study are available on request from the corresponding author as this paper contains information that could compromise the privacy of research participants.
